# EpiMOGA: An Epistasis Detection Method Based on a Multi-Objective Genetic Algorithm

**DOI:** 10.3390/genes12020191

**Published:** 2021-01-28

**Authors:** Yuanyuan Chen, Fengjiao Xu, Cong Pian, Mingmin Xu, Lingpeng Kong, Jingya Fang, Zutan Li, Liangyun Zhang

**Affiliations:** 1 Department of Mathematics, College of Science, Nanjing Agricultural University, Nanjing 210095, China; chenyuanyuan@njau.edu.cn (Y.C.); 2018111006@njau.edu.cn (F.X.); piancong@njau.edu.cn (C.P.); 2 College of Agriculture, Nanjing Agricultural University, Nanjing 210095, China; 2016201007@njau.edu.cn (M.X.); 2017201005@njau.edu.cn (L.K.); 2018201002@njau.edu.cn (J.F.); 2019201003@njau.edu.cn (Z.L.)

**Keywords:** genome-wide association studies, high-order epistasis, genetic algorithms, multi-objective optimization, Alzheimer’s disease

## Abstract

In genome-wide association studies, detecting high-order epistasis is important for analyzing the occurrence of complex human diseases and explaining missing heritability. However, there are various challenges in the actual high-order epistasis detection process due to the large amount of data, “small sample size problem”, diversity of disease models, etc. This paper proposes a multi-objective genetic algorithm (EpiMOGA) for single nucleotide polymorphism (SNP) epistasis detection. The K2 score based on the Bayesian network criterion and the Gini index of the diversity of the binary classification problem were used to guide the search process of the genetic algorithm. Experiments were performed on 26 simulated datasets of different models and a real Alzheimer’s disease dataset. The results indicated that EpiMOGA was obviously superior to other related and competitive methods in both detection efficiency and accuracy, especially for small-sample-size datasets, and the performance of EpiMOGA remained stable across datasets of different disease models. At the same time, a number of SNP loci and 2-order epistasis associated with Alzheimer’s disease were identified by the EpiMOGA method, indicating that this method is capable of identifying high-order epistasis from genome-wide data and can be applied in the study of complex diseases.

## 1. Introduction

A genome-wide association study (GWAS) involves the examination of genetic variations in a given genome to identify genetic variations associated with a phenotype. This type of study has become a powerful tool for detecting single nucleotide polymorphisms (SNPs) and has detected a number of single SNPs associated with complex diseases [1]. However, the analysis of SNPs cannot fully explain the pathogenesis of complex human diseases. After the comprehensive screening of susceptible genes, the cumulative genetic risk of all relevant SNPs is often lower than the susceptibility due to genetics, which is called missing heritability [2]. One possible source of this unaccounted risk is the interaction between multiple SNPs or epistasis. Therefore, it is very important to detect high-order epistasis for the analysis of complex diseases.

However, there are several challenges associated with detecting high-order epistasis on a whole-genome scale. First, detection of high-order epistasis is the process of screening a high-dimensional search space constituted by SNP data, which entails an enormous computational burden. In particular, with the rapid development of high-throughput sequencing technology, a large amount of SNP data has been obtained, resulting in a geometric increase in the computational amount of the detection process. At the same time, the large amount of SNP data also creates a “small sample size problem”: the number of samples contained in a GWAS dataset is much smaller than the number of SNPs [3]. In addition, the diversity of complex disease models also requires that the detection methods can be applied to different situations without potential preference.

In the face of these challenges, a variety of epistasis detection methods have been proposed in recent years, the theoretical basis of which presents the situation of a diversified development of statistics theory, informatics theory, etc. In statistics, logistic regression analysis is the most basic epistasis detection method [4]. Although the detection results are simple and easy to explain, there are obvious shortcomings, such as overfitting and a large amount of calculation. Additional epistasis detection methods based on statistical theory include the multiple functional regression model (MFRG) [5], variance analysis-based method called FastANOVA [6], and the BEAM method based on Bayesian theory [7]. However, the complexity of statistical factors and parameters, low detection efficiency and low accuracy in statistical theory limit its application in high-order epistasis detection. In recent years, information gain, mutual information and the K2 score of the Bayesian network have been widely used. The detection methods also show diversified development. In case-control studies, the epistasis detection methods can be mainly divided into three categories: exhaustive methods, search algorithms and machine learning.

Exhaustive methods verify all possible SNP combinations in the data set, which can effectively avoid the omission of epistasis detection, but require a massive amount of computation. BOOST is a classic multistage exhaustive approach that defines SNP epistasis through logistic regression and divides the entire detection process into search and filter phases [8]. The efficiency of this method is comparatively high, but it is applicable to detecting the interaction between only two SNPs, leading to limited utility. FDHE-IW is a multistage exhaustive method applicable to high-order epistatic detection [9]. In this method, two new indexes, symmetric uncertainty and interaction weight factor based on mutual information and joint entropy, are proposed as criteria for searching and screening.

Search algorithms include stochastic methods and heuristic searches. Stochastic methods are mostly realized by random sampling and a probability calculation. For example, BEAM detects suspected SNPs and their interactions via a Bayesian partitioning model and computes the posterior probabilities of the candidates belonging to true-associated SNPs and epistasis via Markov Chain Monte Carlo (MCMC) sampling [7]. A heuristic search is an approximate search with the guidance of heuristic information, which can effectively reduce the search space and find the optimal solution as soon as possible. This type of search includes the detection method FHSA-SED [10] with the harmony search algorithm and ant colony optimization algorithms MACOED [3], epiACO [11] and AntEpiSeeker [12]. Epi-GTBN is an epistasis mining approach based on a genetic algorithm and the Bayesian network [13] in which a heuristic search strategy applies a genetic algorithm to the Bayesian network and calculates the BIC score to guide the search process and the evaluation index of the Bayesian network.

Machine learning methods are also able to detect the epistasis of SNPs, such as the support vector machine [14], random forest [15], neural network, or association rules [16]. SNPrule is an epistasis detection method based on predictive rule learning [17] that can infer possible higher-order epistasis by identifying the predictive rules contained in epistasis interactions. In the process of machine learning, the requirement of sample size and the amount of calculation on cross-validation limit its application in higher-order epistasis detection. In addition, there are other drawbacks to machine learning, such as difficulty in explaining the results and the tendency of overfitting.

Most of these studies show the potential preference of disease models or perform poorly in small sample size problems. Therefore, this paper proposes a multi-objective epistasis detection method based on a genetic algorithm, called the multi-objective genetic algorithm (EpiMOGA). In this approach, multi-objective optimization is applied to the fitness function in the genetic algorithm and multiple candidate solutions are searched to solve the complex pattern optimization problem. We verified the performance of EpiMOGA in both simulation data and a real dataset and compared the results with some representative methods, including FDHE-IW [9], BOOST [8], Epi-GTBN [13], and SNPrule [17]. Experimental results suggest that EpiMOGA performs robustly in datasets with different characteristics and disease models. Most importantly, this method performs particularly well in datasets with a small sample size.

## 2. Materials and Methods

To compare the performance of the EpiMOGA method in different disease models and characteristics, GAMETES [18] was used to generate 26 simulated datasets. Using 2-order epistasis detection as an example, the performance of EpiMOGA in all simulated datasets was tested and compared with other comparative methods. At the same time, the epistasis detection of the real Alzheimer’s disease dataset was completed using EpiMOGA.

### 2.1. Simulated Datasets

GAMETES is a fast, flexible, and precise tool for generating complex n-locus datasets with random architectures [18]. Genetic constraints and the description of n-order epistasis characteristics were realized through stipulating heritability (h^2^), minor allele frequency (MAF) and prevalence (P(D)). The heritability h2 is the proportion of the population variance due to genetic differences [19], with values ranging from 0 to 1, and can represent the degree to which the expression of a trait is affected by genetic differences. Given the heritability h^2^ and MAF of SNPs, the P(D) of different disease models can be calculated under the assumption of Hardy-Weinberg Equilibrium (HWE). GAMETES can also set other parameters, such as the number of samples in the disease group, the number of samples in the control group, and the number of SNPs.

By setting the heritability, the minor allele frequency and the number of samples, 26 simulation datasets were generated that were divided into two models: disease models with marginal effects (DME) and disease models with no marginal effects (DNME). There are three different DME categories: the multiplication effect model, the threshold effect model and the concrete model [20], which will be called DME model 1, DME model 2, and DME model 3, respectively. Finally, 100 datasets were generated for each parameter setting, with each dataset containing 100 SNPs. More information about the disease models can be found in the Supplementary Materials.

### 2.2. Real GWAS Dataset

To further verify the performance of EpiMOGA, we used it to analyze a real dataset on Alzheimer’s disease. Alzheimer’s disease is a progressive neurodegenerative disease with an insidious onset, which is believed to be a complex disease that is affected by both epistasis and the environment [21]. Its clinical symptoms include memory impairment, cognitive impairment, executive dysfunction, and impairment of visuospatial skills [21]. The pathogenesis of Alzheimer’s disease is related to a variety of factors and a single gene cannot fully explain the high heritability, so it is more meaningful to conduct epistasis detection of Alzheimer’s disease.

We downloaded the genetic data of 305 Alzheimer’s patients and 127 control group cases from the ADNI database (http://adni.loni.usc.edu/). A total of 620,901 SNPs were identified by the platform Illumina human610-quad BeadChip. This is a typical small sample dataset, in which the sample size is far smaller than the number of SNPs.

First, quality control was performed on the dataset. All SNP loci were screened by call ratio, MAF and the Hardy Weinberg equilibrium test. SNP loci with a call ratio less than 0.95, MAF less than 0.05, *p*-value from the Hardy-Weinberg test less than 0.05, or *p*-value from the Chi-squared test less than 0.05 were removed from the AD dataset. For each sample, if the call rate was < 95%, the sample was excluded. Finally, the AD dataset contained 22,164 SNPs and 432 samples, including 305 case samples and 127 control samples.

### 2.3. Problem Description

Let $X=\left\{X_{1},X_{2},\ldots ,X_{N}\right\}$ be a set of SNP variables with N SNP sites of L samples. The homozygous major allele (AA), the heterozygous allele (Aa), and the homozygous minor allele (aa) are denoted by 0, 1, and 2, respectively. Let Y be the phenotypic variable with a value of $\left\{y_{1},y_{2},\ldots ,y_{J}\right\}$. J represents the number of phenotype states Y, which is equal to 2 in the case-control dataset. We denote the control group and the case group as 0 and 1, respectively.

Let $S=\left\{S_{1},S_{2},\ldots ,S_{K}\right\}\left(i<N,S_{i}\in X\right)$ be a K-order epistasis model, where $S_{i}$ is an SNP loci. F(S,Y) is a score function to evaluate the correlation between the K-order epistasis model S and phenotype Y. The smaller magnitudes of F(S,Y) indicate a stronger correlation between the K-SNP model and the phenotype. The high-order epistasis detection problem can be transformed into an optimal solution problem by the score function. The mathematical model of epistasis detection can be expressed as

(1) $$\underset{X}{\min}F\left(S,Y\right),X=\left(X_{S_{1}},X_{S_{2}},\ldots ,X_{S_{K}}\right)$$

where $S_{i}\left(i=1,\;2,\ldots ,K\right)$ represents the *i*–th SNP site, and $X_{S_{i}}$ is the expression value of the ith SNP.

### 2.4. Bayesian Network Scoring and Gini Index

The Bayesian network (BN) model is a probabilistic graph model that can be represented by a directed acyclic graph. The BN model can represent the causal relationship by linking edges between nodes. In a GWAS study, nodes represent genetic variants and disease status, and the conditional dependence relationship between the corresponding nodes can be represented by a set of directed edges (more details about the BN can be found in the Supplementary Materials). Therefore, the BN scoring criterion is also applicable to the scoring function of epistasis detection.

Thus, we choose the K2 score based on the BN scoring criterion [22] as one objective. The K2 score can be used as a measure of correlation; the lower the logarithm score is, the stronger the association between the SNP subset and the disease.

In addition, the Gini index, the diversity index of the binary classification problem, was selected as the other objective. The Gini index (Gini coefficient) is a measure of statistical dispersion, which can be used to measure the impurity of a data partition or the inequality among values of a frequency distribution [23]. The lower the Gini index is, the better the capability of the corresponding SNPs to distinguish between the disease and control groups, which means the stronger the association between the SNP subset and the disease is.

The K2 score and Gini index can be described as:

(2) $$K2-\mathrm{Score}=\prod_{i=1}^{I}\left[\frac{\left(J-1\right)!}{\left(n_{i}+J-1\right)!}\prod_{j=1}^{J}N_{ij}!\right],$$

(3) $$\mathrm{GI}-\mathrm{Score}=\sum_{i=1}^{I}P_{i}\left(1-\sum_{j=1}^{J}P_{i,j}^{2}\right)=\sum_{i=1}^{I}\frac{n_{i}}{n}\left(1-\sum_{j=1}^{J}{\left(\frac{n_{ij}}{n_{i}}\right)}^{2}\right),$$

where *I* is the number of possible combinations of genotypes theoretically possible ($I=3^{K}$ for the K-way SNP combination), $n_{i}$ is the number of cases with the ith genotype combination, $n_{ij}$ is the number of cases where the disease node takes the *j*th phenotype, and $p_{i,j\;}\left(p_{i,j}=n_{ij}/n_{i}\right)$ means the estimated probability of the association between the *i–*th genotype combination and the *j*–th phenotype. It can be seen from the calculation formula of the two scores that the genotype frequency of K-SNP needs to be calculated only once to obtain the K2 score and Gini index, which is conducive to reducing the computational burden.

In the Supplementary Materials, the results of single-objective detection and multi-objective detection are compared through simulation experiments. The results show that the multi-objective method is more suitable for epistasis detection than the single-objective method.

### 2.5. Pareto Optimal Approach

In EpiMOGA, we designated two objectives: the K2 score and the Gini index. The higher-order epistasis detection problem was transformed into a multi-objective optimization problem with an SNP set from GWAS data as the solution space. The mathematical model can be described as:

(4) $$min\left\{\begin{array}{c} f_{1}\left(X_{i}\right)=K2-Score\left(X_{i}\right)\\ f_{2}\left(X_{i}\right)=GI-Score\left(X_{i}\right) \end{array}\right\},$$

However, in practice, it is difficult to achieve the optimal solution of both objectives. In general, the situation will be that the solution has better performance on one objective and may perform worse on the other compared with other solutions. Therefore, the problem of multi-objective optimization usually does not seek the unique optimal solution but is transformed into finding the nondominated solution through the Pareto optimal approach. Suppose $X_{1}$ and $X_{2}$ are two solutions of the multi-objective problem. If one of the following conditions is satisfied:

(1) $f_{1}\left(X_{1}\right)<f_{1}\left(X_{1}\right)\&\&f_{2}\left(X_{1}\right)<f_{2}\left(X_{2}\right)$,
(2) $f_{1}\left(X_{1}\right)=f_{1}\left(X_{1}\right)\&\&f_{2}\left(X_{1}\right)<f_{2}\left(X_{2}\right)$,
(3) $f_{1}\left(X_{1}\right)<f_{1}\left(X_{1}\right)\&\&f_{2}\left(X_{1}\right)=f_{2}\left(X_{2}\right)$,

the solution $X_{1}$ is said to dominate the solution $X_{2}$. The relationship between the two solutions has only two possibilities: either one dominates the other or neither dominates [3]. If solution $X_{1}$ was not dominated by other solutions, $X_{1}$ could be called a nondominant solution. The nondominant solution set is the candidate set that we want to search.

### 2.6. EpiMOGA

The EpiMOGA proposed in this paper is a multi-objective heuristic high-order epistasis detection method, and the whole process can be divided into two parts: search and screening. On the one hand, to maintain better performance in different sample sizes and solve the small sample size problem, we applied a heuristic algorithm to the epistasis search process. Using a genetic algorithm instead of a general exhaustive search can speed up the search process and reduce the computational complexity. On the other hand, to reduce the potential bias and adapt to different disease models, the Pareto optimal method was applied to the fitness function of the genetic algorithm to achieve multi-objective optimization.

A genetic algorithm is a random search algorithm based on the survival of the fittest principle and the genetic mechanism of Darwinian evolution [24]. Compared with the traditional optimization method, a genetic algorithm includes multiple possible solutions in the initial population and performs genetic operations and evaluation on multiple solutions in each iteration. This method can effectively reduce the risk of falling into local optimization and has good global search ability, which is widely used in combinatorial optimization problems. Therefore, the results of a genetic algorithm are dependent on the quality of the initial population. In the EpiMOGA method, the range of the initial solution was expanded by searching several times, and different candidate subsets of the output were screened to reduce this dependence.

**Figure 1:**
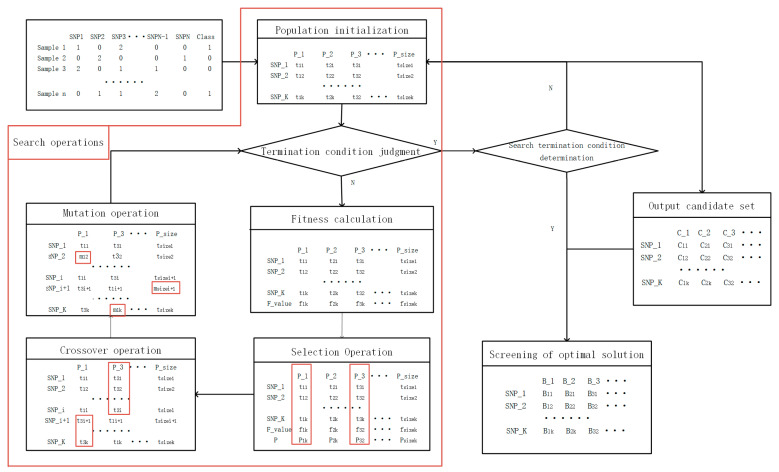
Flow chart of the EpiMOGA algorithm.

Figure 1 is the flow chart of the EpiMOGA algorithm and Algorithm 1 is the pseudocode of the EpiMOGA algorithm. The method can be divided into the following steps: population initialization, genetic operations (selection, crossover, and variation) and screening.
**Algorithm 1.** EpiMOGA pseudocode **Input:**
Num: the number of the initial population, positive integers greater than 1 X: m × n matrix consisted of 0 and 1, representing the states of m samples at n SNP sites. Y: 1 × m vector consisted of 0 and 1, representing the state of m samples. Maxtimes:Maximum number of iterations **Output:** Best: the set of optimal SNP combinations01: begin 02: **For** i = 1:Num 03:  Initial population_i 04:  Evaluate population:fitvalue⬅Twoobjection(population_i,X,Y) 05:   **while** (generation <Maxtimes) 06:   Selection 07:   Crossover 08:   Mutation 09:   Evaluate population 09:   Output candidate chromosome 10:  **end** 11: **end** 11: Merge candidate set 12: Evaluate candidate set 13: Output Best⬅best SNP combinations 14: **end**

#### 2.6.1. Encoding Schemes, Initializing the Population

Encoding is the process of transforming the parameters of a problem space into chromosomes or individuals with a certain structure in the genetic space. Encoding schemes should meet the requirements of completeness, soundness and nonredundancy. General chromosome encoding methods include binary encoding, real encoding, and character encoding. Based on the large number of SNPs, the real encoding method was selected in the EpiMOGA algorithm. A K-SNP was represented by K real numbers, which was the order of SNP loci in the GWAS dataset. The chromosome encoded by K-SNP and the initialized population can be expressed as:

(5) $$X_{i}=\left(S_{i1},S_{i2},\ldots ,S_{iK}\right),POP=\left(X_{1},X_{2},\ldots ,X_{P_{s}}\right),$$

where $X_{i}$ is the *i*–th chromosome in the genetic algorithm and $S_{it}\left(t=1,\;2,\ldots ,K\right)$ means the order of the SNP in the GWAS dataset, with values ranging from 1 to N. POP is a $K\times P_{s}$ matrix, representing the initial population, where $P_{s}$ is the size of the initial population.

The process of population initialization consists of generating random numbers in the range of 1 to N that represent SNP sites in the corresponding order.

#### 2.6.2. Genetic Operations

Genetic operations include selection, crossover and mutation, respectively simulating the biological phenomena of natural selection, biological reproduction and gene mutation. The search process of the genetic algorithm was guided by the iteration of the genetic operation.

##### Selection Operation

The selection operation in a genetic algorithm is a process of natural selection based on fitness. In each generation, the fitness of each individual in the population is evaluated, and multiple individuals are stochastically selected from the current population (based on their fitness) [24]. Common selection operators include roulette wheel selection, stochastic tournament, and expected value selection. EpiMOGA mainly uses the roulette selection method, which is a playback random sampling method. The probability $P_{i}$ of $X_{i}$ being selected is calculated using the following equation:

(6) $$P_{i}=\frac{f_{i}}{\sum_{i=1}^{P_{s}}f_{i}},$$

where $f_{i}$ is the fitness value of chromosome i and $P_{s}$ is the size of the population.

##### Crossover Operation

The crossover operation in a genetic algorithm is a process in which a pair of chromosomes randomly exchange some loci according to certain rules to form two new individuals. Common crossover operators include one-point crossover, uniform crossover, and arithmetic crossover. The EpiMOGA algorithm mainly uses one-point crossover, which is suitable for real number encoding. When the random number (ranging from 0 to 1) is less than the crossover probability Pc, the crossover point is selected randomly, and part of the chromosomes of the paired individuals are exchanged at this point (see the following operation).

(7) $$\begin{array}{c} X_{i}=(S_{i1},S_{i2},\ldots ,S_{iK})\\ X_{j}=(S_{j1},S_{j2},\ldots ,S_{jK}) \end{array}\overset{\mathrm{cpoint}\;=\;\mathrm{round}(\mathrm{rand}*K)}{\to }\begin{array}{c} {X_{i}}^{\prime }=(S_{i1},S_{i2},\ldots ,S_{iC_{po\mathrm{int}}},S_{jC_{po\mathrm{int}}+1},\ldots ,S_{jK})\\ {X_{j}}^{\prime }=(S_{j1},S_{j2},\ldots ,S_{jC_{po\mathrm{int}}},S_{iC_{po\mathrm{int}}+1},\ldots ,S_{iK}) \end{array},$$

where $X_{i},X_{j}$ represents a pair of chromosomes, $X_{i}^{’},X_{j}^{’}$ represents new chromosomes formed by crossover, and $C_{po\mathrm{int}}$ is a randomly selected point.

##### Mutation Operation

The mutation operation in a genetic algorithm refers to the process in which some gene loci in the individual coding string are replaced with other values according to certain rules to form new chromosomes. Common mutation operators include simple mutation, uniform mutation, and boundary mutation. The EpiMOGA algorithm mainly uses simple mutation. When the random probability is less than the mutation probability Pm, the value of a certain gene locus or a certain number of loci is randomly assigned for the mutation operation (see the following operation):

(8) $$\begin{array}{c} X_{i}=(S_{i1},S_{i2},\ldots ,S_{iK})\overset{M_{po\mathrm{int}}\;=\;\mathrm{ceil}(\mathrm{rand}*K)}{\to }{X_{i}}^{\prime }=(S_{i1},S_{i2},\ldots ,S_{iM_{po\mathrm{int}}},\ldots ,S_{iK})\\ S_{iM_{po\mathrm{int}}}=ceil(rand*N), \end{array}$$

where $X_{j}$ represents a chromosome, $X_{j}$ represents a new chromosome formed by the mutation operation and $M_{po\mathrm{int}}$ represents the variation point.

In the process of population initialization and genetic operation, it should be noted that a new chromosome should be tested to determine whether the following two conditions are met: there were no duplicated SNPs in the chromosome and no duplicated chromosomes in the population. Only chromosomes that meet these criteria can be preserved in the population.

##### 2.6.3. Fitness Function

In the optimized genetic algorithm, different individuals in the population (candidate solutions) can be divided into two sets without superposition according to the Pareto optimal method: a dominant solution set and a nondominant solution set. The fitness function generally uses the numerical form to evaluate the merits of a solution. The higher the fitness value is, the better the solution. Therefore, in the EpiMOGA method, the nondominant solution was regarded as the better individual and the fitness value was determined according to the number of solutions that it dominated. Furthermore, the fitness value of the dominant solution was uniformly set to 1. These is the pseudocode of the fitness function calculation in EpiMOGA in Algorithm 2.
**Algorithm 2.** Twoobjection() pseudocode **Input:** X: m × n matrix consisted of 1 and 0, representing the states of m samples at n SNP sites. Y: 1 × m vector consisted of 0 and 1, representing the state of m samples. Pop:t × K matrix, represents t K-SNP combinations **Output:** Objvalue: 1 × t vector, representing the fitness value of t K-SNP combinations, which is a positive integer greater than or equal to 1.01: **begin** 02: Initialization: objvalue(1:pm) = 2 03: **For** i = 1:t 04:  [objvalue1(i), objvalue2(i)] = TwoScore01(X(:,pop(i,:)),y); 05: **end** 06: **For** each i, j = 1:t 07: If((objvalue1(j)<objvalue1(i))&&(objvalue2(j)<objvalue2(i)))||((objvalue1(j)<objvalue1(i)) &&(objvalue2(j)==objvalue2(i)))||((objvalue1(j)==objvalue1(i))&&(objvalue2(j)<objvalue2(i))) 08:     objvalue(j) = objvalue(j) + objvalue(i); 09:     objvalue(i) = 1; 10:     break; 11:    **end** 12: **end**

##### 2.6.4. Screening Candidate Sets

The screening of candidate sets was also based on the K2 score and the Gini score. According to the Pareto optimal method, the candidate sets obtained from multiple searches were divided into nondominant solution sets and dominant solution sets. Among them, the nondominant solution set was output as the elite set and became the final result of the EpiMOGA method.

## 2.7. Evaluation Criteria

In a GWAS, we generally regard that disease-related SNP combinations are positive and disease-unrelated SNP combinations are negative, leading to an imbalanced problem in the GWAS dataset exemplified by the existence of a much larger number of negatives than positives. For example, a simulated dataset that is made up of 100 SNPs can produce $C_{100}^{2}=4950$ different 2-SNP combinations. In these combinations, only one of them is truly associated with disease, indicating positive, and all other 4949 are negative. Therefore, more appropriate indicators should be selected to evaluate the quality of detection results.

In the simulation experiment, we choose Power and F_measure to evaluate the performance of the algorithm from two aspects, detection efficiency and detection accuracy. The specific definition is as follows:

1. Power measures the ability of algorithms to detect functional SNP combinations from all datasets, which is used for comparing the detection efficiency of different methods. The specific calculation is as follows:

(9) $$Power=\frac{D_{T}}{D},$$

where D is the number of datasets and $D_{T}$ denotes the number of datasets that accurately identify functional SNP combinations.

2. The F_measure is a comprehensive index based on recall and precision. Recall, also known as sensitivity, measures the identification ability of positive samples by calculating the proportion of true positive outputs in the total positive output. In addition, precision calculates the proportion of true positive outputs in the total output. Since the total number of results is not taken into account in the calculation of recall but is related to the value of precision, there is a contradiction between recall and precision in that enhancing recall by increasing the total number of results may cause a drop in precision. The F_measure is the harmonic mean of recall and precision and reflects the detection accuracy of a method. The calculation formula is as follows:

$$recall=\frac{TP}{TP+FN},$$

$$precision=\frac{TP}{TP+FP},$$

$$F\_measure=\frac{2}{1/precision+1/recall}$$

## 3. Results

### 3.1. Simulation Experiments and Results

#### 3.1.1. Parameter Setting

In the simulation experiment, to compare the performance of the methods in different situations, we designed 3 simulation experiment cases that included 26 simulation datasets. Taking 2-order epistasis detection as an example, EpiMOGA was compared with other comparative epistasis detection methods (including Epi-GTBN, BOOST, SNPrule and FDHE-IW) in terms of detection efficiency given by Power and detection accuracy given by the F_measure. Parameter adjustment was unnecessary in the BOOST method. In SNPRuler, we set the listSize to 2000, depth to 2 and updateRatio to 0.5. Population size was the common important parameter in both EpiMOGA and Epi-GTBN. Therefore, we set the population size to 50 in these methods to ensure a fair comparison. Similarly, the probability of variation and the probability of crossover were set as 0.01 and 0.6, respectively. The number of the initial population Num is a unique parameter of EpiMOGA that was set as 60 after a series of simulation experiments (details of the experiments can be found in the Supplementary Materials).

#### 3.1.2. Simulation Experiment Case 1

**Figure 2:**
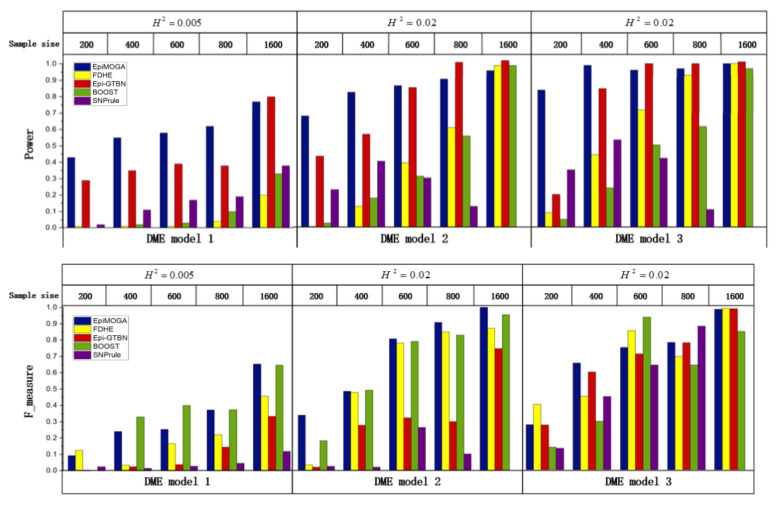
Detection efficiency and accuracy comparisons between EpiMOGA and other methods on 3 DME models.

We used the three DME introduced in the materials section to generate 15 datasets with sample sizes of 200, 400, 600, 800 and 1600 when MAF = 0.1, which were used to analyze the influence of the disease model and sample size on the performance of methods. Figure 2 shows the detection efficiency in terms of Power and accuracy in terms of the F_measure of the different methods in the 15 simulation datasets.

In the DME1 dataset with a sample size of 400, the detection accuracy of EpiMOGA was 0.09 lower than that of the BOOST method, but the detection efficiency of EpiMOGA was more than ten times that of the BOOST method. Therefore, we can see that EpiMOGA is obviously more suitable for this dataset compared to the BOOST method.

At the same time, EpiMOGA also performs well in datasets with large sample sizes. Campared with Epi-GTBN, we found that if two method have similar value on one objective, Epi-GTBN is more likely to be worse than EpiMOGA on the other. As an example, for the DME2 dataset with a sample size of 800, the two index values of EpiMOGA and Epi-GTBN are shown in Table 1.

In Table 1, we can see that the detection efficiency of EpiMOGA was slightly lower than that of Epi-GTBN, but it also reached 90%. Moreover, the detection accuracy of EpiMOGA is three times that of Epi-GTBN. Therefore, it is reasonable to believe that EpiMOGA is more suitable and performs better than Epi-GTBN on this dataset.

In summary, compared with other methods, EpiMOGA significantly optimized the detection performance on small datasets of three different disease models. In larger sample sizes, EpiMOGA maintained a good and stable detection performance.

#### 3.1.3. Simulation Experiment Case 2

**Figure 3:**
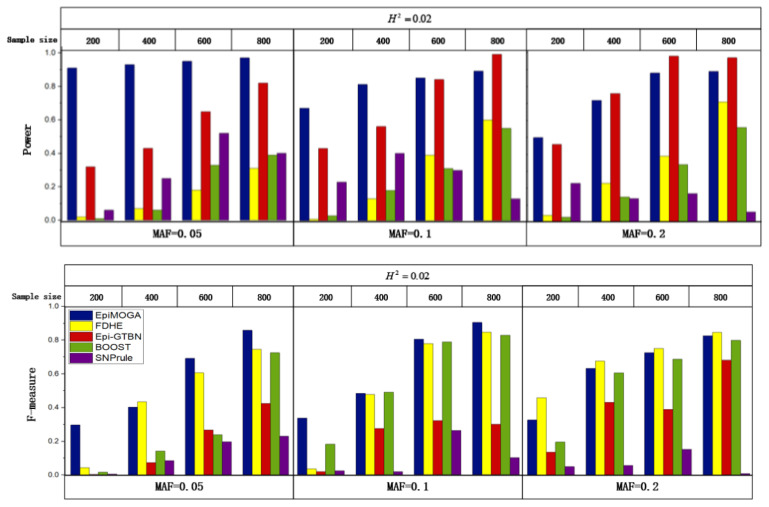
Detection efficiency and accuracy comparisons between EpiMOGA and other methods on the DME2 model with different MAF and sample sizes.

In the same disease model DME2, 12 simulation datasets with MAF set to 0.05, 0.1, and 0.2 and the sample size set to 200, 400, 600, and 800 were generated to compare the performance of different characteristics. Figure 3 shows the detection efficiency and accuracy of different methods in 12 simulation datasets.

In Figure 3, we can see that the performance of EpiMOGA in small datasets is still superior to that of other methods with the change in MAF. At the same time, EpiMOGA performed very well on the 4 datasets with MAF = 0.05 and was far superior to other methods in both evaluation indexes. In other datasets, the evaluation index of EpiMOGA was obviously better than that of SNPrule. On all datasets, the F_measure of FDHE-IW and BOOST was similar to that of EpiMOGA, but these methods were far lower than EpiMOGA on Power. We can also see that the Power of EpiMOGA was equivalent to that of Epi-GTBN, but EpiMOGA was obviously better on the F_measure. As an example, on the dataset with a sample size of 600 and MAF = 0.2, the two indexes of EpiMOGA, FDHE-IW and Epi-GTBN are shown in Table 2.

In Table 2, we can see that FDHE-IW and Epi-GTBN were slightly better than the EpiMOGA method on one objective and far lower than EpiMOGA on the other objective. To conclude, EpiMOGA performed better in both detection efficiency and detection accuracy, especially for small sample datasets.

#### 3.1.4. Simulation Experiment Case 3

**Figure 4:**
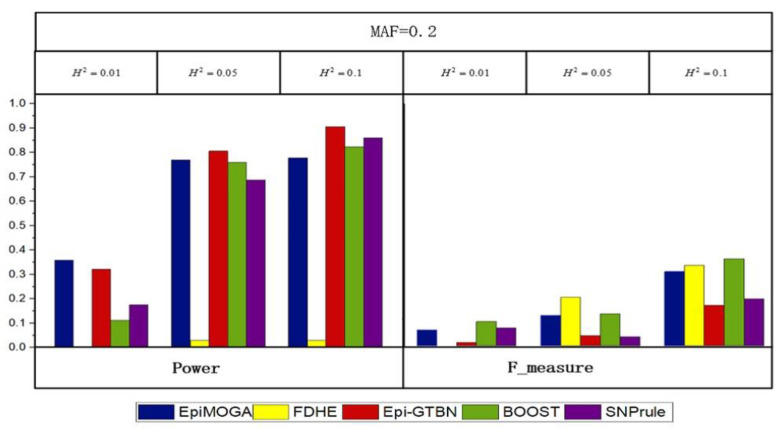
Detection performance comparisons between EpiMOGA and other comparative methods on DNME models with 3 different parameters.

Three simulation datasets with an MAF of 0.2 and heritability of 0.01, 0.05, and 0.1 were generated to compare the performance of different methods on DNME. Figure 4 shows the detection efficiency and accuracy of the different methods.

In Figure 4, we can see that the performance of these methods was similar, except for FDHE-IW. Compared with the results of the DME models, SNPrule performed significantly better on the DNME models, while the opposite occurred in FDHE-IW because of the potential preference for the disease model. This phenomenon did not exist in the EpiMOGA method.

We also have a simulation experiments case about the number of SNP loci and the details about this experiments case can be found in Supplementary Materials.

### 3.2. Real Experiment and Results on the Alzheimer’s Disease Dataset

**Figure 5:**
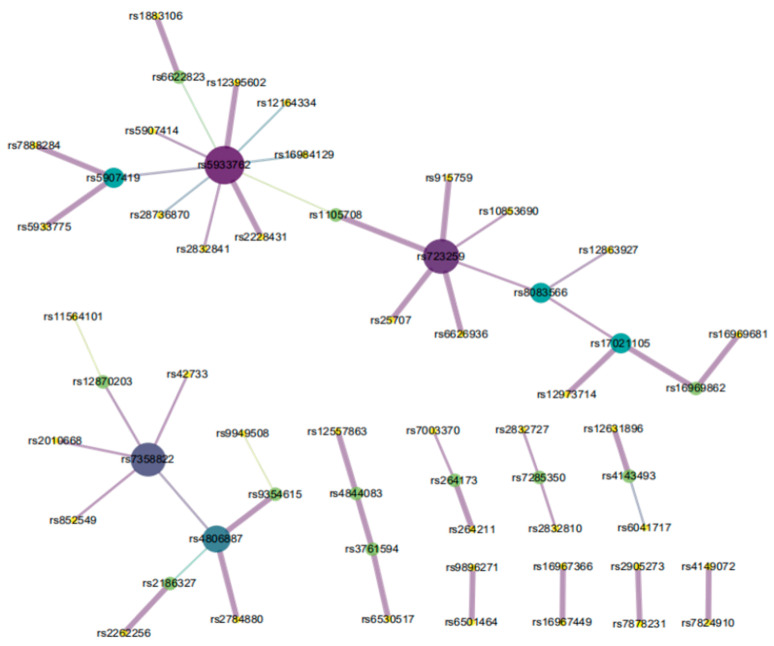
SNP-SNP network of AD.

The EpiMOGA method was used to perform 2-order epistasis detection on Alzheimer’s disease data. After searching and filtering by the EpiMOGA method, 89 2-order SNP pairs were output as the final result. In further analysis, Chi-squared tests were performed on 89 pairs of SNPs; if their *p*-values were less than 0.001, SNP combinations were retained. Subsequently, a total of 48 pairs of SNPs were obtained, including 58 SNP loci. Figure 5 shows the final generated network diagram using Cytoscape [25].

In Figure 5, each dot represents an SNP site and the line between the two dots represents a 2-SNP. The larger area and the darker color of the dot mean that there are the more SNP sites are connected. Similarly, the stronger association between 2-SNP and Alzheimer’s disease, the thicker a line is.

Among the 58 SNP loci, 31 SNPs were in the coding region and 27 SNPs were in the noncoding region. Some of genes have been reported to be related to Alzheimer’s disease. For example, rs42733 is an A/G SNV variation in the coding cyclic adenosine monophosphate (*cAMP*) response element binding protein (*CREB*) 5 gene. *CREB* signaling plays a major role in long-term memory formation. Defective *CREB* signaling underlies impaired hippocampal neurogenesis and cognitive deficits in Alzheimer’s disease [26]. rs16984129 is an SNP loci in the *ARMCX5-GPRASP2* gene, which is also known as *P60TRP*. The protein coded by this gene was initially identified to be downregulated in the temporal lobe of brains in Alzheimer’s disease patients [27] and has been described as a potential target for the development of strategies for inhibiting the early signaling mechanisms involved in neurodegenerative diseases such as AD [28]. rs9949508 is located on the *DLGAP1* gene, which is also called *GKAP* or *SAPAP1*, and rs6530517 is a G/T SNV variation in the FRMPD4 gene, while encoded protein FRMPD4 (FERM and PDZ domain-containing 4) is a neural scaffolding protein [29]. Both of these proteins interact with PSD-95 [30]. Important variations in the distribution pattern of PSD-95 represent a marker in AD and contribute to functional deterioration by impairing the neuronal network [31]. rs11564101 is a G/A/T/C SNV variation in the mitoguardin (*MIGA*) 2 gene, which is a mitochondrial protein encoding gene. Experiments show that the loss of a MIGA leads to mitochondrial defects and neurodegeneration and results in fragmented mitochondria [32]. Mutations in core members of the mitochondrial fission/fusion machinery are responsible for severe neurodegenerative diseases, such as Alzheimer’s disease. rs12631896 is an SNP loci in the *CACNA2D3* gene. Studies have shown that *CACNA2D3* is associated with cognitive ability and intelligence and is a predisposing risk factor for Alzheimer’s disease [33].

Several genes are also related to the clinical symptoms of Alzheimer’s disease. rs7358822 is an SNV variation in the *ATP8A2* gene. *ATp8A2* is a known coding gene associated with complex neurological diseases and has high mRNA expression in hippocampal neurons [34]. Mutations in the *ATP8A2* gene have been reported to cause severe recessive neurological diseases in humans, characterized by encephalopathy, intellectual disability, cerebellar atrophy and optic atrophy [35]. rs7285350 is located in the LARGE1 gene region. *LARGE* was found to be a component of the AMPA-type glutamate receptor (AMPA-R) protein complex, a main player for learning and memory in the brain [36]. Mutations in the human *LARGE* gene result in severe intellectual disability and affect learning and memory, which is consistent with the clinical symptoms of Alzheimer’s disease. rs12395602 is a variant loci of the gene *CNKSR2. CNKSR2* is highly expressed in the brain and is known to play a role in synaptogenesis [37]. Absent *CNKSR2* causes intellectual, attention, and language deficits [38], which are consistent with the clinical manifestations of Alzheimer’s disease. rs25707 is located on the gene FMR1. Studies have shown important roles of FMRP in synaptic plasticity and proper functioning of the neural network [39]. This genetic change causes a risk for the development of neurodegenerative diseases [40]. In addition, rs17021105 is located on the *GRID2* gene. The coding protein *GRID2* is considered to be a suppressor in neurodegeneration [41]. Therefore, it is reasonable to surmise that rs7358822, rs7285350, rs12395602, rs25707 and rs17021105 are correlated with Alzheimer’s disease.

In addition, rs12870203 is a variant on the *MTUS2* gene, which is also called *CAZIP*. One study suggested a role of *CAZIP* in the development and function of the nervous system in vertebrates [42]. rs2186327 and rs2262256 are located in the coding region of the *TIAM-1* gene. *TIAM-1* has been implicated in the development of the central nervous system [43] and contributes to neurite extension in defined neuronal populations [44]. The decay of neurons is also a cause of neurodegenerative diseases. rs28736870 is an SNV variation in the *GTPBP6* gene. One study showed that the overexpression of GTPBP6 is negatively correlated with speech cognition and has a causal relationship with neural development and speech function [45]. rs5933762, rs723259 and rs5933775 are SNP variants located in the coding region of the *SHROOM2* gene. The influence and clinical phenomenon of *SHROOM2* missense have not been found in the existing literature. In the test results, *SHROOM2* showed an epistatic role with other Alzheimer’s disease-related genes. Therefore, we speculate that *MTUS2*, *GTPBP6*, *TIAM-1* and *SHROOM2* are likely associated with Alzheimer’s disease.

Table 3 lists the top ten 2-SNP combinations with *p*-values from Chi-squared tests, including SNP loci information, *p*-value of a single SNP site, *p*-value of 2-SNP and the prediction accuracy of the support vector machine (SVM). By comparing the *p*-values, we can see that the *p*-value of 2-SNP was far less than that of a single SNP, indicating that epistasis does exist. Moreover, we can see from the last column that the SVM prediction accuracies of these 2-SNP combinations were approximately 70%, which may be worth study for biologists. (Additional information about the 3-order epistasis detection on Alzheimer’s disease data can be found in the Supplementary Materials).

In summary, the results of EpiMOGA detection in the dataset on Alzheimer’s disease were reliable and had a certain biological significance.

## 4. Discussion

A genome-wide association analysis is mainly used to detect the correlation between a single SNP locus and a phenotype [1]. The results of a GWAS cannot explain the lack of heritability because of ignoring the interaction between genes (epistasis), which limits its ability to analyze complex diseases. Epistatic detection can help us better explore the occurrence of complex diseases and have a positive significant impact on the prevention, discovery and treatment of complex diseases. In this paper, we proposed a multi-objective epistasis detection method named EpiMOGA.

EpiMOGA is an epistasis detection method that completes a spatial search through a genetic algorithm and carries out screening based on a multi-objective function. The K2 score based on the BN criterion [22] and the Gini index of diversity of the binary classification problem [23] were used as the search and screening objectives. Through the Pareto optimal approach, the epistasis detection problem was extended to find a nondominated set of solutions. In the genetic algorithm, the individuals in the population were divided into two parts, the dominant solution set and the nondominant solution set, and the fitness value of the nondominant solution set was determined according to the number of the dominant solution. After several iterations of the genetic operation, the optimal solution was obtained as the candidate set. Due to the dependence of the genetic algorithm on the initial population, as well as the randomness of the genetic operation, the candidate set obtained by a single search could not guarantee global optimization. Therefore, EpiMOGA performed several search operations to obtain multiple independent candidate solution sets, in which the nondominant solutions were selected as the final detection result.

Experimental results in simulated datasets and an Alzheimer’s disease dataset showed that EpiMOGA is an effective method for epistasis detection. Compared with other comparative methods, EpiMOGA can maintain good detection performance with balanced detection efficiency and detection accuracy in datasets with different characteristics and disease patterns. At the same time, EpiMOGA also shows a strong advantage in small datasets, in which both of the evaluation criteria are obviously optimized. Under the dilemma of a small sample size problem caused by a large number of SNP data, EpiMOGA has a more extensive application.

EpiMOGA also has some limitations, such as the dependence on parameters. In EpiMOGA, increasing the number of searches can effectively reduce the impact of the initial population and the risk of local optimization. However, as the number of searches increases, the run time also increases. In simulation experiment case 2, we observed that the detection efficiency of EpiMOGA remained above 90% but showed a decreasing trend with the increase in MAF. One reason for this result is the effect of the parameter settings on the efficiency of the method. Therefore, to improve the performance of EpiMOGA, it is necessary to select different and appropriate parameters when detecting epistasis in different datasets.

In the analysis of the dataset on Alzheimer’s disease, we found that a number of SNP sites in the noncoding genomic region were recognized. The function of SNP in the noncoding genomic region should be a part of future research, as it can be conducive to research on complex diseases. The other important future direction is determining how to add prior knowledge to the method to accelerate the search process.

## Figures and Tables

**Table 1:** Two index values of EpiMOGA and Epi-GTBN in the DME2 dataset (sample size = 800).

Method	Power	F-Measure
EpiMOGA	0.90	0.9072
Epi-GTBN	0.99	0.3004

**Table 2:** Two index values of EpiMOGA, FDHE-IW, BOOST and Epi-GTBN (MAF = 0.2, sample size = 600).

Method	Power	F-Measure
EpiMOGA	0.88	0.7273
FDHE-IW	0.39	0.7529
BOOST	0.34	0.6887
Epi-GTBN	0.98	0.3925

**Table 3:** Top-10 epistatic interactions associated with AD.

Order	SNP1	P1	SNP2	P2	P	SVM
1	rs17021105	1.52934 × 10^−7^	rs8083566	3.48624 × 10^−5^	1.30237 × 10^−9^	0.720265781
2	rs7003370	1.93263 × 10^−6^	rs264173	0.00032777	2.73286 × 10^−8^	0.694629014
3	rs2832810	6.48301 × 10^−5^	rs7285350	0.040037803	2.91614 × 10^−8^	0.694518272
4	rs852549	0.001864809	rs7358822	0.000190734	2.06066 × 10^−7^	0.706256921
5	rs2010668	0.00234868	rs7358822	0.000190734	2.77707 × 10^−7^	0.706256921
6	rs2832727	0.000632929	rs7285350	0.040037803	3.38987 × 10^−7^	0.701605759
7	rs8083566	3.48624 × 10^−5^	rs723259	0.01579838	3.40313 × 10^−7^	0.73654485
8	rs10853690	3.48624 × 10^−5^	rs723259	0.01579838	3.40313 × 10^−7^	0.73654485
9	rs42733	0.025710022	rs7358822	0.000190734	5.87876 × 10^−7^	0.706256921
10	rs2832841	5.07116 × 10^−5^	rs5933762	0.000330143	7.53923 × 10^−7^	0.727131783

Note: In the table, P1 is the *p*-value of the Chi-square test on SNP1, P2 is the *p*-value of the Chi-square test on SNP2, and P is the *p*-value of the Chi-square test on this 2-SNP combination.

## Data Availability

The data presented in this study are available in https://github.com/ycfszd1996/EpiMOGA.

## Supplementary Materials

The following are available online at https://www.mdpi.com/2073-4425/12/2/191/s1, Figure S1: K-SNP epistasis Bayesian network model, Figure S2: Detection efficiency and accuracy comparisons between EpiMOGA and the single-objective method, Figure S3: A line diagram of the standardized values of the Power and time, Figure S4: Detection efficiency comparisons between EpiMOGA and other comparative methods on DME models with 5 different SNP number, Figure S5: The detect time of EpiMOGA and Epi_GTBN on different datasets, Figure S6: SNP-SNP network of 3-order epistasis detection, Table S1: Penetrance functions of the three DME epistasis models, Table S2: Penetrance tables of the DME and DNME epistasis models with different sets of parameters, Table S3: Top-10 3-order epistasis interactions associated with AD. All the simulate data and code can be found here: https://github.com/ycfszd1996/EpiMOGA.
