# Supplementary material for: EpiMOGA: An Epistasis Detection Method Based on a Multi-Objective Genetic Algorithm
Source: Genes (Basel). 2021 Jan 28;12(2):191. doi: 10.3390/genes12020191 (PMC7911965; doi:10.3390/genes12020191)
Supplement: Supplementary file 1 [file genes-12-00191-s001.pdf]

Supplementary Material

1. Bayesian network scoring criteria and Gini scoring criteria

1.1. Bayesian network

The Bayesian network (BN) model is a probabilistic graph model that can be represented by a directed acyclic graph. In the Bayesian network, a set of variables is denoted as a set of nodes and a set of edges represents their conditional dependencies. BN provides a way of expressing causality between variables, which has a natural virtue in expressing the relationship between SNPs and disease. It has been widely used in studying genomic data.

Let $X = \{X_1, X_2, ..., X_N\}$ be a set of SNP variables and Y be the phenotypic variable. If there exists a directed edge from Xi to Y, we call Xi the parent node of Y. Suppose that $P = \{P(X_i \mid pa(X_i))\}$ is the conditional probability set of BN. The joint probability distribution for an N-node BN can be represented as:

$$P(X_1, X_2, ..., X_N) = \prod_{i=1}^{N} P(X_i \mid pa(X_i))$$

As the affections of SNPs in the causative of disease rather than the affections among SNPs are concerned about, we can build a particular two-layer BN to represent the causative relation in a GWAS study, where one layer consists of a set of SNP nodes and the other of the disease node[3]. An example of exhaustive 2-SNP epistasis BN models in a set of SNP X can be seen in Supplementary Figure 1.

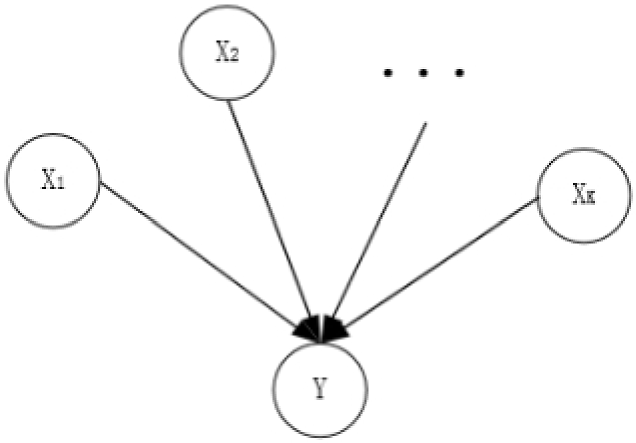

Supplementary Figure 1. K-SNP epistasis Bayesian network model

1.2. K2 score

Let M represents a Bayesian network and D be a sample dataset that contains values of SNPs and phenotype states. The Bayesian score computes the posterior probability of the BN model structure given the GWAS data, denoted as P(M|D). Through Bayes' theorem as follows, we can associate P(M|D) with P(D|M):

$$P(M \mid D) = \frac{P(D \mid M)P(M)}{P(D)} \quad (1)$$

where P(D|M) is the class-conditional density, P(M) is the prior probability of model, and P(D) represents the probability of data D, which is a constant for all Bayesian network models based on dataset D[46]. The equation above can be written as

$$P(M \mid D) \propto P(D \mid M)P(M) \quad (2)$$

P(D|M) can be calculated as follows:

$$P(D \mid M) = \prod_{i=1}^{I} (\frac{\Gamma(\alpha_i)}{\Gamma(n_i + \alpha_i)} \prod_{j=1}^{J} \frac{\Gamma(n_{ij} + \alpha_{ij})}{\Gamma(\alpha i_j)}) \quad (3)$$

where I is the number of genotype combinations and J is the number of phenotype states Y, n_i is the number of cases with SNP nodes taking the ith combination, n_{ij} is the number of cases when the disease node takes the jth state and his parents take the ith combination, and α_{ij} is the hyperparameter of the model referring to the Dirichlet distribution and indicates the prior belief about the number of cases when the nodes take the corresponding ith combination and jth state [3,22], and $\alpha_i = \sum \alpha_{ij}$.

Therefore, we can set all hyperparameters $\alpha_{ij} = 1$, which indicates an equal likelihood for all possible distributions in each BN model, and set the prior probability as a constant, meaning that we take the same possibility for all models in a set of SNP X. Moreover, P(M|D) can be computed as follows:

$$P(M \mid D) \propto \prod_{i=1}^{I} (\frac{(J-1)!}{(n_i + J - 1)!} \prod_{j=1}^{J} n_{ij}!) \quad (4)$$

The Bayesian score turns out to be the following K2 score:

$$K2\text{-}Score = \prod_{i=1}^{I} \left(\frac{(J-1)!}{(n_i + J - 1)!} \prod_{j=1}^{J} n_{ij}! \right) \quad (5)$$

1.2. Gini index

The Gini index (Gini coefficient) is a measure of statistical dispersion that can be used to measure the impurity of a data partition or the inequality among values of a frequency distribution [23]. For a binary classification case-control problem, the Gini index is a diversity index which is defined as

$$GI - Score = \sum_{i=1}^{I} P_i (1 - \sum_{j=1}^{J} P_{i,j}^2 \ ) \tag{6}$$

where P_{ij} is the estimated probability that the i-th genotype combination actually associated with phenotype y_j, $(1 - \sum_{j=1}^{J} P_{ij}^{\ 2})$ is the estimated probability that genotype combination is misclassified as phenotype y_j, and P_i is the percentage of the i-th genotype combination in sample set.

2. Comparison of single-objective method and multi-objective method

To prove the suitability of the multi-objective method in epistasis detection, we used the genetic algorithm with the K2 score and Gini score as a single target to detect 2-SNP epistasis in simulated datasets, compared with EpiMOGA. All parameters of the genetic algorithm were set the same. Taking the results of the dataset with the DME model 2 with a sample size of 400 as an example, the Power and F_measure of different methods are shown in Supplementary Figure 2.

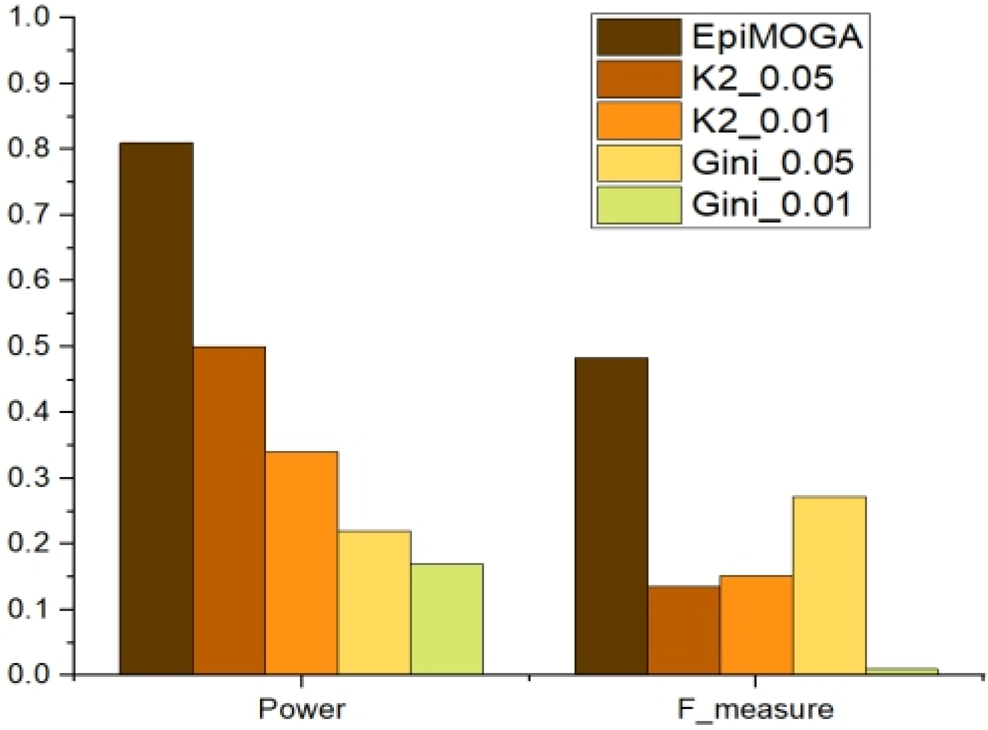

Supplementary Figure 2. Detection efficiency and accuracy comparisons between EpiMOGA and the single-objective method

In Supplementary Figure 2, we can find that the performance of EpiMOGA is significantly superior to other single-objective methods, indicating that the multi-objective model can obviously improve the effect and is suitable for epistasis detection.

In addition, the general single-objective detection method can be divided into two parts: search and screen. In the process of screening, it is important to select a threshold in the method. A threshold method that is overly strict will lead to the reduction of detection efficiency Power, while detection accuracy F_measure will decrease if the threshold method is too broad. For example, there are different results with threshold values of 0.01 and 0.05 in Fig. S2. We can find that the Power of single-target detection is enhanced but the detection accuracy F_measure is decreased with the increase of the threshold. Thus, the advantage of the MOGA multi-objective approach is that threshold selection is not required in the whole process since it has been replaced by the Pareto optimal approach.

3. The epistasis model

Simulation datasets are generated by GAMETES, which describes different disease models by the parameters of the genetic heritability (h^2) and the minor allele frequency (MAF). The genetic heritability h^2 is defined as follows:

$$h^2 = \frac{\sum_{i=1}^{3^j} P(G_i)(P(D \mid G_i) - P(D))^2}{P(D)(1 - P(D))} \tag{7}$$

where $P(G_i)$ is the frequency of genotype combinations that can be solved under the assumption of Hardy-Weinberg Equilibrium (HWE). P(D) is the disease prevalence that can be computed by the following equation:

$$P(D) = \sum_{i=1}^{3^j} P(G_i) P(D \mid G_i) \tag{8}$$

Disease models with marginal effects (DME) include three different categories.

Model 1 is a 2-locus multiplicative model in which the conditional probability P(D|Gi) increases multiplicatively with the incremental presence of the disease allele.

Model 2 is a 2-locus threshold model in which the P(D|Gi) does not increase until the number of disease alleles passes the threshold.

Model 3 is a 2-locus concrete model used to mimic the effect that epistasis has on susceptibility to handedness and the color of swine[47,48].

Penetrance functions of the three DME epistasis models can be seen in Table S1. In the table, the parameters are determined by the given disease prevalence P(D), the genetic heritability h2 and the MAF.

Supplementary Table 1. Penetrance functions of the three DME epistasis models

Model 1		Loci 1		
		AA	Aa	aa
Loci 2	BB	α	α	α
	Bb	α	α(1+β)2	α(1+β)3
	bb	α	α(1+β)3	α(1+β)4
Model 2		Loci 1		
		AA	Aa	aa
Loci 2	BB	α	α	α
	Bb	α	α(1+β)	α(1+β)
	bb	α	α(1+β)	α(1+β)
Model 3		Loci 1		
		AA	Aa	aa
Loci 2	BB	α	α(1+β)	α(1+β)
	Bb	α(1+β)	α	α
	bb	α(1+β)	α	α

Supplementary Table 2. Penetrance tables of the DME and DNME epistasis models with different sets of parameters

DME Model 1	P(D)=0.1,h2=0.005,MAF=0.1			DME Model 2	P(D)=0.1,h2=0.02,MAF=0.1		
	AA	Aa	aa		AA	Aa	aa
BB	0.0960	0.0960	0.0960	BB	0.0918	0.0918	0.0918
Bb	0.0960	0.1971	0.2824	Bb	0.0918	0.3192	0.3192
bb	0.0960	0.2824	0.4047	bb	0.0918	0.3192	0.3192
DME Model 3	P(D)=0.1,h2=0.02,MAF=0.1			DME Model 2	P(D)=0.1,h2=0.02,MAF=0.05		
	AA	Aa	aa		AA	Aa	aa
BB	0.0717	0.1636	0.1636	BB	0.0958	0.0958	0.0958
Bb	0.1636	0.0717	0.0717	Bb	0.0958	0.5331	0.5331
bb	0.1636	0.0717	0.0717	bb	0.0958	0.5331	0.5331
DNME Model 1	h2=0.01,MAF=0.2			DME Model 2	P(D)=0.1,h2=0.02,MAF=0.2		
	AA	Aa	aa		AA	Aa	aa

BB	0.2216	0.2758	0.1414	BB	0.0836	0.0836	0.0836
Bb	0.2587	0.1690	0.4013	Bb	0.0836	0.2099	0.2099
bb	0.2781	0.1279	0.4196	bb	0.0836	0.2099	0.2099
DNME Model 2	h2=0.05,MAF=0.2			DNME Model 3	h2=0.1,MAF=0.2		
	AA	Aa	aa		AA	Aa	aa
BB	0.4988	0.6388	0.7649	BB	0.2278	0.0742	0.2121
Bb	0.6665	0.3887	0.0831	Bb	0.0726	0.7758	0.1540
bb	0.5432	0.5265	0.9533	bb	0.2407	0.0251	0.0518

Simulation experiment case 1 used three different DME disease models and generated 15 datasets with sample sizes of 200, 400, 600, 800 and 1600 at the same MAF and P(D).

Simulation experiment case 2 used the same disease model (DME Model 2) and generated 12 data sets with sample sizes of 200, 400, 600 and 800 at an MAF of 0.05, 0.1, and 0.2. Part of these datasets are the same as the dataset of case 1.

Simulation experiment case 3 was an experiment for three datasets of disease models with no marginal effects (DNME), with MAF=0.2, H2 set to 0.01, 0.05 and 0.1 and sample size =600.

4. The parameter of EpiMOGA

Num is a specific parameter of EpiMOGA that denotes the number of the initial population. In general, genetic algorithms, the quality of the initial population has an important impact on the result. In the EpiMOGA method, the range of the initial population was expanded with the increase of Num, and different candidate subsets of the output were screened to reduce the dependence between the quality of the initial population and the results of method. Therefore, the increase of Num can improve the detection efficiency of the method, but the detection time will also increase at the same time.

To study the influence of different parameter sizes on method results, we set Num as 20, 40, 60, 80 and 100 for epistasis detection on the same simulated dataset. Supplementary Figure 3 is a line diagram of the standardized values of the detection Power and the time.

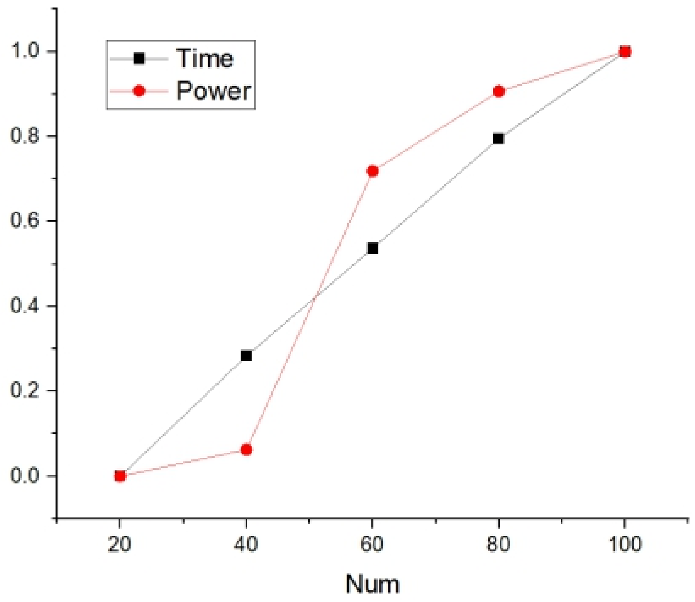

Supplementary Figure 3. A line diagram of the standardized values of the Power and time

As shown in the figure, the time increases at a similar rate as the size of Num increases. At the same time, the growth rate of detection efficiency in terms of Power is the greatest in the stage of Num from 40 to 60. Therefore, we choose Num=60 in EpiMOGA.

5. Analysis on the influence of SNP quantity on detection efficiency

The number of SNP sites is also one of the important factors affecting the performance of methods. We designed a simulation experiment to analyze the influence of the number of SNP sites on different methods. Without changing other parameters, we used the DME model 2 to generate 5 datasets with SNP number of 100, 200, 300, 400 and 600 when sample size is 600. Supplementary figure 4 shows the detection efficiency of the different methods on these five datasets.

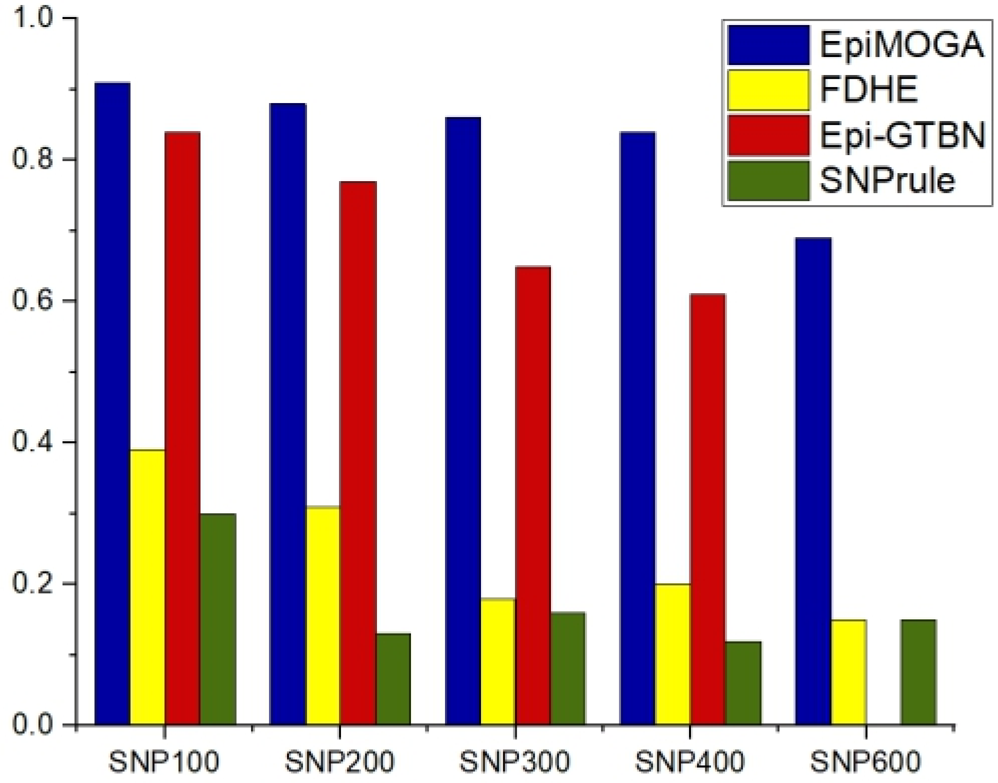

Supplementary Figure 4. Detection efficiency comparisons between EpiMOGA and other comparative methods on DME models with 5 different SNP number.

In supplementary Figure 4, as the number of SNP loci increase, the difficulty of detection increases significantly, and the detection efficiency of all methods has reduced. However, the detection efficiency of EpiMOGA on almost all datasets is better than FDHE-IW and SNPrule. Although Epi-GTBN has maintained a relatively good detection efficiency, the detection time has increased rapidly, which will take a long time to complete the detection of the SNP600 and SNP800 datasets. We compared the detection time of EpiMOGA and Epi-GTBN on different datasets, as shown in supplementary Figure 5.

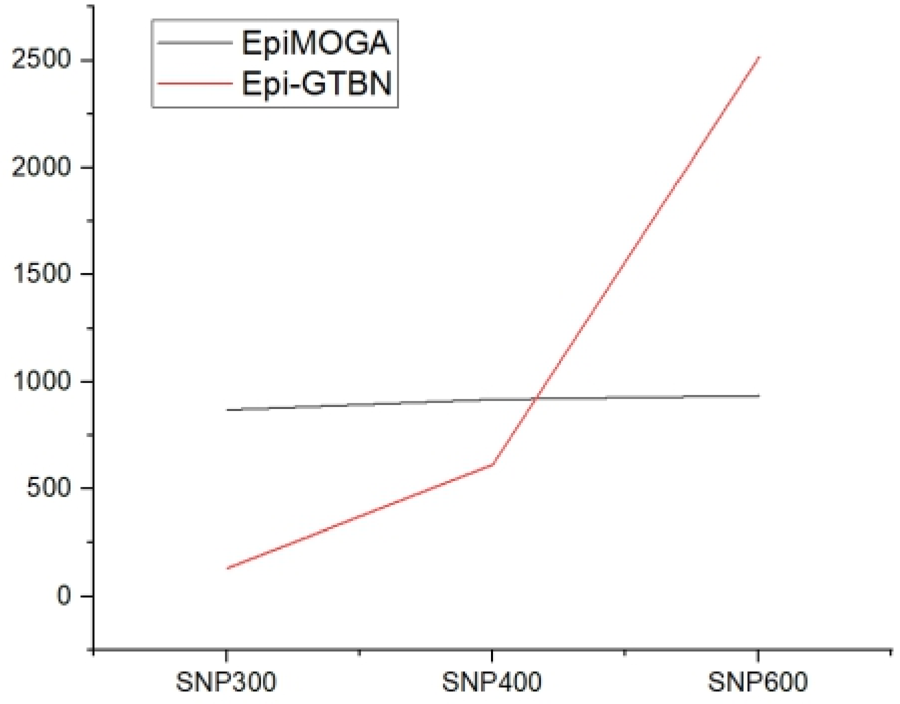

Supplementary Figure 5. The detect time of EpiMOGA and Epi_GTBN on different datasets.

In supplementary Figure 5, the abscissa represents the datasets with different number of SNP loci, and the ordinate is the detection time of a single dataset in seconds. As shown on the figure, the detection time of EpiGTBN is positively correlated with the number of SNPs. The detection time of Epi_GTBN exceeds 40 minutes on a SNP600 single dataset, and it will takes at least 3 days to complete the detection of all 100 datasets. At the same time, we also find that the detection time of Epi_GTBN on a single dataset of 800SNP is more than 90 minutes, what means that Epi_GTBN will take at least 7 days to complete all tests. In comparison, the detection time of EpiMOGA has increased slowly, maintaining a relatively stable state. On the SNP600 dataset, the detection efficiency of EpiMOGA almost reaches 70%, which is even better than the detection result of Epi_GTBN on SNP400 dataset. Meanwhile, the detection time of a single SNP600 dataset is only one third of that of Epi_GTBN.

Therefore, it is reasonable to believe that EpiMOGA is more suitable for the detection on a large number of SNP loci datasets than other methods.”

6. The results of 3-order epistasis detection on the Alzheimer's disease dataset

The EpiMOGA method was used to perform 3-order epistasis detection on the Alzheimer's disease dataset. After searching and filtering by the EpiMOGA method, 111 3-order SNP pairs were output as the final result. In further analysis, Chi-squared tests were performed on 111 pairs of SNPs; if their p-values were less than 0.01, SNP combinations were retained. Subsequently, a total of 43 pairs of SNPs were obtained, including 80 SNP loci. Among the 80 SNP loci, 37 SNPs were in the coding region and 43 SNPs were in the noncoding region.

Supplementary Figure 6 shows the final generated network diagram using Cytoscape [25]. Furthermore, Supplementary Table 4 lists the top ten 3-SNP combinations with p-values from Chi-squared tests, including SNP loci information, p-value of a single SNP site, p-value of 2-SNP and the prediction accuracy of the support vector machine (SVM).

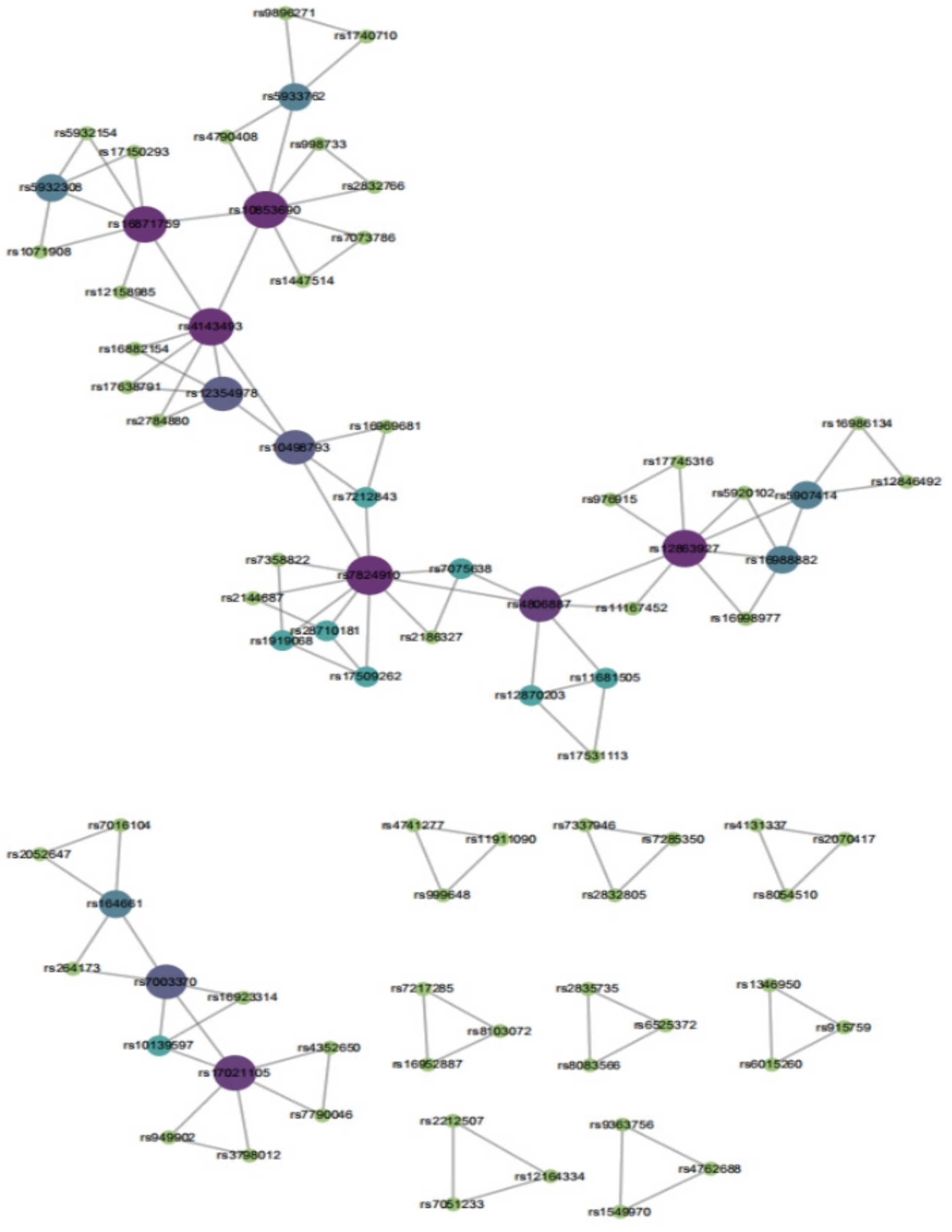

Supplementary Figure 6. SNP-SNP network of 3-order epistasis detection.

Supplementary Table 3. Top-10 3-order epistasis interactions associated with AD.

SNP1			SNP2			SNP3			P	SVM
name	Gene	P1	name	Gene	P2	name	Gene	P3		

rs2052647	NA	0.022754092	rs7016104	NA	0.023533506	rs164661	EXTL3	0.000795134	1.92455E-29	0.860354374
rs4741277	NA	0.002546555	rs999648	NA	0.044808863	rs11911090	WDR4	0.041087596	3.08477E-24	0.857751938
rs17021105	GRID2	1.52934E-07	rs3798012	SEMA5A	0.004119354	rs949902	PACRG	1.34311E-05	5.38706E-13	0.697009967
rs11681505	NA	0.001822551	rs17531113	NA	0.008965881	rs12870203	MTUS2	0.000676362	7.20917E-10	0.706423034
rs28710181	CALML6	0.020928358	rs7824910	NA	0.007080722	rs17509262	NA	0.000276904	8.96284E-10	0.704097453
rs10498793	PKHD1	0.002319041	rs16969681	NA	0.006310536	rs7212843	NA	0.014261099	2.884E-09	0.704097453
rs7073786	PCDH15	0.002377489	rs1447514	SLC17A6	0.002544484	rs10853690	NA	3.48624E-05	7.95237E-09	0.692524917
rs11681505	NA	0.001822551	rs12870203	MTUS2	0.000676362	rs4806887	ZNF57	0.007080722	2.94215E-08	0.706423034
rs4143493	PKHD1	0.002319041	rs12354978	KIAA1217	0.005787059	rs2784880	NA	0.02382676	4.33987E-08	0.704097453
rs4131337	NA	0.049852716	rs8054510	NA	0.033069097	rs2070417	TIAM1	0.004160467	1.44555E-07	0.73648948

Note: In the table, P1 is the p-value of the Chi-square test on SNP1, P2 is the p-value of the Chi-square test on SNP2, P3 is the p-value of the Chi-square test on SNP2, and P is the p-value of the Chi-square test on this 3-SNP combination. The ‘Gene’ value of the SNP in the noncoding region is NA.

In Supplementary Table 3, the genes GRID2, MTUS2 and TIAM_1, which have been analyzed in the 2-SNP test results, are associated with Alzheimer's disease. In addition, KIAA1217 has been reported to be related to Alzheimer's disease. MiR-603, a novel primate-specific miRNA and an intronic miRNA of a human brain highly expressed gene KIAA1217, is implicated in the risk and pathogenesis of Alzheimer's disease [49]. SLC17A6 (solute carrier family 17 member 6) is also known as VGLUT2. Experiments showed that a significant decrease in VGLUT2 was observed in Alzheimer's disease and the VGLUT2 reduction was correlated with the degree of cognitive impairment, assessed with the clinical dementia rating (CDR) score [50]. It can be used as a valuable neurochemical marker of dementia in Alzheimer's disease. PACRG (Parkin Co-Regulated Gene) is a gene that shares a bidirectional promoter with the gene Parkin that can result in neurodegeneration [51,52]. Furthermore, PACRG plays an important role in the central nervous system [52]. SEMA5A (Semaphorin 5A) is a transmembrane semaphorin that has been found to control axon guidance during neural development [53]. It was previously reported to be a susceptibility locus for neurodegenerative disease and for autism [54,55].

The EXTL3 gene has been identified as a receptor molecule for regenerating islet-derived (REG) protein ligands [56]. Overexpression of EXTL3 increases the effect of recombinant Reg-1α on neurite outgrowth [57]. Studies have shown that Reg-1α is overexpressed during the very early stages of Alzheimer disease, and Reg-1α deposits were detected in the brains of patients with Alzheimer disease [57]. WDR4 is the human ortholog of the yeast Trm82, an essential component of the Trm8/Trm82 holoenzyme [58]. Mutations in the human WDR4 gene result in severe intellectual disability and affect speech and language, which is consistent with the clinical symptoms of Alzheimer's disease [58,59]. Therefore, it is reasonable to surmise that these genes are related to Alzheimer's disease.

By comparing the p-values in Supplementary Table 3, we can see that the p-value of 3-SNP was far less than that of a single SNP, indicating that epistasis does exist. Moreover, we can see from the last column that the SVM prediction accuracies of these 3-SNP combinations were approximately 70%, and the maximum is over 86%. In summary, the results of EpiMOGA 3-order epistasis detection on the dataset of Alzheimer's disease were reliable and had a certain biological significance.
